# Supplementary figures and images for: Trends in hepatocellular carcinoma and viral hepatitis treatment in older Americans
Source: PLoS One. 2024 Nov 1;19(11):e0307746. doi: 10.1371/journal.pone.0307746 (PMC11530004; doi:10.1371/journal.pone.0307746)

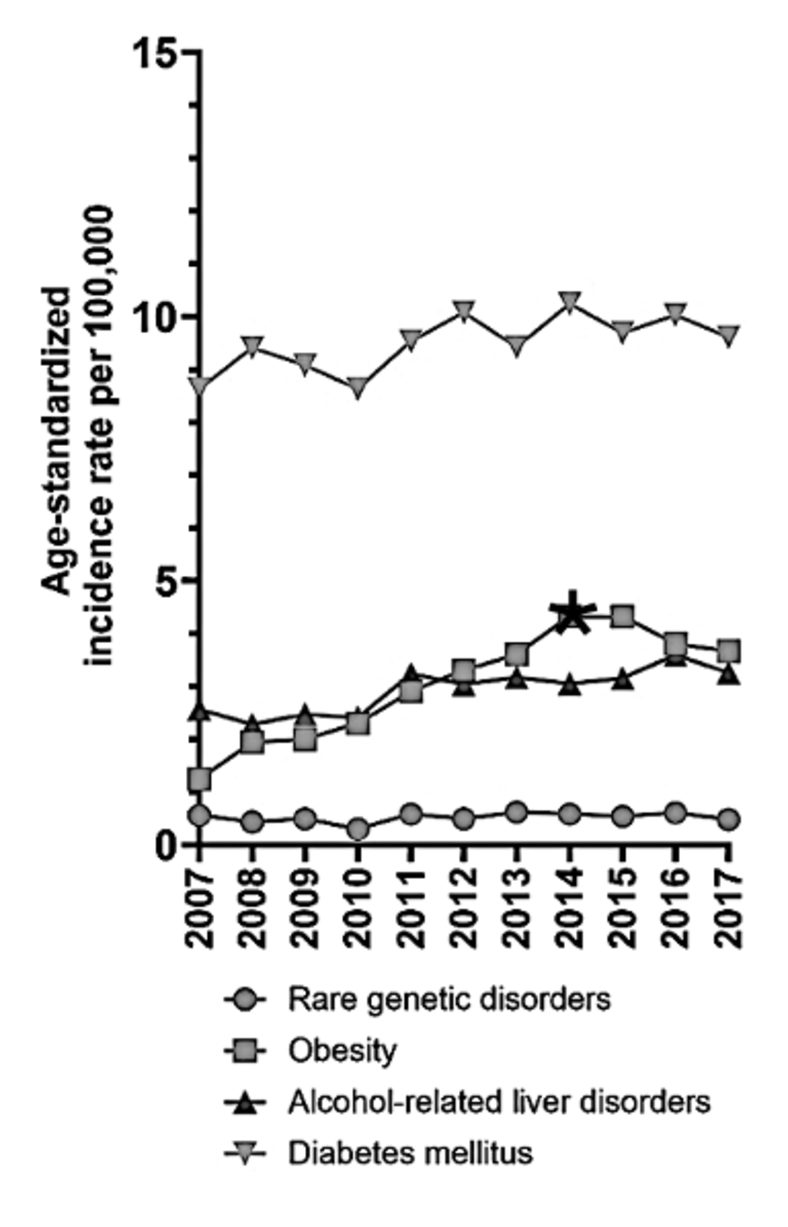

Supplement: S1 Fig — The occurrence of risk factors is not mutually exclusive. (TIF) [file pone.0307746.s001.tif]
