# Supplementary material for: Trends in hepatocellular carcinoma and viral hepatitis treatment in older Americans
Source: PLoS One. 2024 Nov 1;19(11):e0307746. doi: 10.1371/journal.pone.0307746 (PMC11530004; doi:10.1371/journal.pone.0307746)
Supplement: S1 Table — (DOCX) [file pone.0307746.s002.docx]

S1 Table. Timeline of FDA approval of HBV treatments

| **FDA approval** | **Type of therapy** | **Generic**  **Name** | **Brand Name** | **Indication** | **Manufacturer** |
| --- | --- | --- | --- | --- | --- |
| July 1992 | interferon-based | interferon alpha-2b | Intron A | chronic hepatitis B in patients 1 year of age or older with compensated liver disease | Schering |
| December 1998 | nucleoside analogs | lamivudine | Epivir HBV | chronic hepatitis B associated with hepatitis B viral replication and active liver inflammation | GlaxoSmithKline |
| September 2002 | nucleotide analog prodrugs | adefovir dipivoxil | Hepsera | chronic hepatitis B in patients 12 years of age | Gilead Sciences |
| March 2005 | nucleoside analogs | entecavir | Baraclude | chronic hepatitis B virus infection with evidence of active viral replication | Bristol-Myers Squibb |
| May 2005 | interferon-based | pegylated interferon | Pegasys | treatment of adult patients with HBeAg positive and HBeAg negative chronic hepatitis B who have compensated liver disease and evidence of viral replication and liver inflammation | Roche |
| October 2006 | nucleoside analogs | telbivudine | Tyzeka | chronic hepatitis B in adult patients with evidence of viral replication and either evidence of persistent elevations in serum aminotransferases (ALT or AST) or histologically active disease | Novartis |
| August 2008 | nucleotide analog prodrugs | tenofovir | Viread | chronic hepatitis B in adults. | Gilead Sciences |
| November 2016 | Reverse transcriptase inhibitor | Tenofovir alafenamide | Vemlidy | Gilead Sciences | November 2016 |
