# Supplementary material for: Trends in hepatocellular carcinoma and viral hepatitis treatment in older Americans
Source: PLoS One. 2024 Nov 1;19(11):e0307746. doi: 10.1371/journal.pone.0307746 (PMC11530004; doi:10.1371/journal.pone.0307746)
Supplement: S2 Table — (DOCX) [file pone.0307746.s003.docx]

S2 Table. Timeline of FDA approval of HCV treatments

| **FDA approval** | **Generic**  **Names** | **Brand Name** | **Indication** | **Manufacturer Name** |
| --- | --- | --- | --- | --- |
| June 1986 | interferon alpha-2a | Roferon | treatment of chronic hepatitis C in patients 18 years of age or older | Roche |
| March 1997 | interferon alpha-2b | Intron A | treatment of chronic hepatitis C in patients 18 years of age or older with compensated liver disease who have a history of blood or blood-product exposure and/or are HCV antibody positive | Schering |
| August 2001 | pegylated interferon | Pegintron | treatment of adults with chronic hepatitis C virus infection who have compensated liver disease and have not been previously treated with interferon alpha | Schering |
| December 2002 | ribavirin | CoPegus | use in combination with Pegasys or with Roferon for the treatment of adults with chronic hepatitis C virus infection who have compensated liver disease and have not been previously treated with interferon alpha | Roche |
| July 2003 | ribavirin | Rebetol | use in combination with Pegintron for treatment of chronic hepatitis C in patients with compensated liver disease who have not been previously treated with interferon alpha and are at least 18 years of age | Schering |
| July 2010 | interferon aphacon-1 | Infergen | treatment of chronic hepatitis C in patients 18 years of age or older with compensated liver disease who have anti-HCV serum antibodies and/or HCV RNA | Three Rivers Pharma |
| May 2011 | boceprevir | Victrelis | treatment of chronic hepatitis C genotype 1 infection, in combination with peginterferon alfa and ribavirin, in adult patients (18 years of age and older) with compensated liver disease, including cirrhosis, who are previously untreated or who have failed previous interferon and ribavirin therapy. | Merck & Co. |
| May 2011 | telaprevir | Incivek | in combination with peginterferon alfa and ribavirin, for the treatment of genotype 1 chronic hepatitis C (CHC) in adult patients with compensated liver disease, including cirrhosis, who are treatment-naive or who have been previously treated with interferon-based treatment | Vertex Pharmaceuticals |
| October 2013 | sofosbuvir | Sovaldi | treatment of CHC as a component of a combination antiviral regimen | Gilead Sciences |
| November 2013 | simeprevir sodium | Olysio | treatment of CHC genotype 1 infection as a component of a combination antiviral treatment regimen | Janssen Pharmaceuticals |
| October 2014 | ledipasvir/sofosbuvir | Harvoni | a fixed-dose combination of ledipasvir, a hepatitis C virus (HCV) NS5A inhibitor, and sofosbuvir, an HCV nucleotide analog NS5B polymerase inhibitor, and is indicated for the treatment of chronic hepatitis C (CHC) genotype 1 infection in adults. | Gilead |
| December 2014 | ombitasvir, paritaprevir, ritonavir tablets co-packaged with dasabuvir | Viekira Pak | use with or without ribavirin for the treatment of patients with genotype 1 chronic HCV infection including those with compensated cirrhosis | AbbVie Inc. |
| July 2015 | 12.5 mg ombitasvir, 75 mg paritaprevir, 50 mg ritonavir. | Technivie [discontinued on 1 January 2019] | AbbVie Inc. | July 2015 |
| July 2015 | daclatasvir | Daklinza | Bristol-Myers Squibb | July 2015 |
| January 2016 | Elbasvir and grazoprevir | Zepatier | Merck | January 2016 |
| June 2016 | Sofosbuvir and velpatasvir | Epclusa | Gilead Sciences | June 2016 |
| July 2017 | sofosbuvir, velpatasvir and voxilaprevir | Vosevi | Gilead Sciences | July 2017 |
| August 2017 | Glecaprevir, pibrentasvir | Mavyret | AbbVie Inc. | August 2017 |
